# How Homes Are Wrecked

**Published:** 1889-09

**Authors:** 


### HOW HOMES ARE WRECKED.

A brief dispatch in the *Daily News* throws another high light on a social evil which, while it works untold misery, is almost entirely disregarded or ignored. A prominent citizen of Kansas City applies for a divorce from his wife and the mother of his children on the ground of confirmed and incurable drunkenness. The unfortunate woman admits the justice of the action, and only pleads in extenuation that she "contracted the love for liquor by taking it at first as a medicine."

The physician who so prescribed it has a heavy account to render at some bar, either here or hereafter. And there are numbers of others equally culpable in every community. In Chicago hundreds of homes have been desolated through this medical crime, which is not limited to prescribing whisky, but all forms of stimulants and intoxicants—either chloral, hasheesh, morphine, bromidia, etc.

The dipsomaniac is bad enough, but the slave of the opium habit or chloral is infinitely worse. There is nothing too degrading, no trick or art which human ingenuity can invent, no crime, even, too monstrous, tc which the devotees of these infernal drugs will not resort to obtain the stimulant, narcotic, or intoxicant. And in immensely the greater proportion they have been led to their terrible fate by the prescription of a family doctor.

A writer in the September number of the *Popular Science Monthly*, describing how the opium habit is acquired, suggests certain means of preventing the spread of that form of this social evil. We would extend his suggestion so as to include alcoholic liquor as well as opium, and require that no prescription calling for any of this class of agents should be filled more than once by a druggist without having the doctor specially renew the prescription. This would undoubtedly do much to check the spread of these enslaving and insidious habits.—*Chicago News.*

